# Newborn follow-up after discharge from a tertiary care hospital in the Western Cape region of South Africa: a prospective observational cohort study

**DOI:** 10.1186/s41256-017-0057-4

**Published:** 2018-01-12

**Authors:** Jean Paul Muambangu Milambo, KaWing Cho, Charles Okwundu, Abiola Olowoyeye, Leonidas Ndayisaba, Sanjay Chand, Mark H. Corden

**Affiliations:** 1 African Cancer Institute, Stellenbosch University, Cape Town, South Africa; 2 Division of General Pediatrics, Department of Pediatrics, Children’s Hospital Los Angeles, Los Angeles, CA USA; 3 Centre for Evidence Based Healthcare, Stellenbosch University, Cape Town, South Africa; 4 Department of Respiratory Intensive Care, Groote Schuur Hospital, Cape Town, South Africa; 5 Division of Hospital Medicine, Department of Pediatrics, Children’s Hospital Los Angeles, 4650 Sunset Blvd, MS 94, Los Angeles, CA 90027 USA; 6 Department of Pediatrics, Keck School of Medicine, University of Southern California, Los Angeles, CA USA

**Keywords:** Follow-up study, Hospital nursery, Low-income population, Newborn, Patient discharge, Pediatrics, South Africa

## Abstract

**Background:**

Current practice in the Western Cape region of South Africa is to discharge newborns born in-hospital within 24 h following uncomplicated vaginal delivery and two days after caesarean section. Mothers are instructed to bring their newborn to a clinic after discharge for a health assessment. We sought to determine the rate of newborn follow-up visits and the potential barriers to timely follow-up.

**Methods:**

Mother-newborn dyads at Tygerberg Hospital in Cape Town, South Africa were enrolled from November 2014 to April 2015. Demographic data were obtained via questionnaire and medical records. Mothers were contacted one week after discharge to determine if they had brought their newborns for a follow-up visit, and if not, the barriers to follow-up. Factors associated with follow-up were analyzed using logistic regression.

**Results:**

Of 972 newborns, 794 (82%) were seen at a clinic for a follow-up visit within one week of discharge. Mothers with a higher education level or whose newborns were less than 37 weeks were more likely to follow up. The follow-up rate did not differ based on hospital length of stay. Main reported barriers to follow-up included maternal illness, lack of money for transportation, and mother felt follow-up was unnecessary because newborn was healthy.

**Conclusions:**

Nearly 4 in 5 newborns were seen at a clinic within one week after hospital discharge, in keeping with local practice guidelines. Further research on the outcomes of this population and those who fail to follow up is needed to determine the impact of postnatal healthcare policy.

## Background

Early discharge of healthy newborns from the postnatal wards is common in many parts of the world [1, 2]. Early discharge is defined as a discharge from the hospital within 24 h of a vaginal delivery or in two days after a caesarean section. The impact of shorter length of stay on maternal-newborn health is not clear. Some studies favor early discharge, others found no significant difference, and some demonstrated an increased risk for newborn readmission [3–7]. Appropriate follow-up has been recommended to mitigate the morbidity and mortality that may be associated with early discharge [8–11].

The first newborn follow-up visit plays a vital role in bridging care following the immediate newborn period in the hospital. Aside from establishing a relationship with a medical home, the purpose of the follow-up visit is to evaluate for signs/symptoms of infection, jaundice, feeding difficulties, and other issues that may manifest after the first few days of life. Follow-up is generally recommended within two days after discharge for shorter lengths of stay (less than 48 h) up to the first few days following discharge for more prolonged perinatal hospitalizations [8, 9, 12]. When adhered to, such discharge guidelines appear to decrease the likelihood of hospital readmission [13, 14] and may allow for the detection of cases of neonatal sepsis in a region where the incidence is close to 10% [15].

In South Africa, healthy newborns are typically discharged with their mothers within 24 h after an uncomplicated vaginal delivery and the second day following an uncomplicated caesarean section delivery. Upon discharge, mothers are advised to bring their newborns for a follow-up evaluation at their local clinic within one week [16]. The proportion of newborns who follow up at the recommended time after hospital discharge in this region of South Africa is not yet known. The purpose of this study was to determine the number of newborns who have a follow-up visit within seven days and factors associated with failure to follow up in that time frame.

## Methods

### Study design and setting

We conducted a prospective observational cohort study from November 2014 to April 2015 in the postnatal wards at Tygerberg Hospital in Cape Town, South Africa. Tygerberg Hospital is a tertiary hospital that serves the Western Cape region of South Africa, which is home to 5.8 million inhabitants, with 75% living in the metropolitan area of Cape Town. Tygerberg Hospital primarily serves patients of low socioeconomic status without medical insurance [17]. The hospital attends roughly 7500 deliveries per year, a high percentage of which are to mothers referred from regional midwife obstetric units or secondary healthcare facilities for maternal or pregnancy-related conditions that cannot be managed at these facilities [18]. Mothers were considered to have a high-risk pregnancy if they had one or more of the following conditions: anemia, gestational diabetes, HIV, hypertension, or preeclampsia. Other conditions that may have been referred due to referring health unit concerns were not captured with our questionnaire.

### Sample size/data collection strategy

Convenience sampling was used to recruit mother-newborn dyads on day of discharge from the postnatal wards at Tygerberg Hospital. Newborns who were admitted to the neonatal intensive care unit (NICU), had a congenital malformation, were <34 weeks gestational age, or born weighing <1800 g were excluded from the study population. Mothers who were non-English speaking, had no means of telephone contact, or had a discrepancy in their discharge dates compared to their newborns were also excluded.

### Variable measures

Prior to discharge, demographic and medical information from the mother-newborn dyads were collected. A directed questionnaire was used to collect maternal demographic information while data on maternal factors, including mode of delivery, maternal medical conditions and/or obstetric complications (e.g. gestational hypertension, diabetes, pre-eclampsia/eclampsia, urgent caesarean section), neonatal factors (sex, gestational age, birthweight), and length of stay were obtained from review of the medical records. Mothers were contacted by phone between seven and ten days after hospital discharge and asked if a newborn follow-up visit had taken place. Follow-up was defined as a visit to any type of healthcare facility to verify the health of the newborn. Those who had yet to attend a clinic visit within seven days after hospital discharge were asked if barriers existed that prevented follow-up. Participants who could not be reached on the first attempt were contacted again on two other occasions before being considered lost to follow-up and excluded from the final analysis.

### Data analysis

Basic descriptive statistics were used to examine the frequency distribution of all variables. Bivariate logistic regression was used to determine the relationship between the predictor variables and the outcome variable (follow-up visit within seven days after being discharged from the hospital). Multivariable logistic regression was used to examine unique effects from all statistically significant predictors (*P* < 0.05) and predictors approaching statistical significance (*P* < 0.10), which were entered into the model simultaneously. Other items were dropped as a group, and a likelihood ratio test comparing the full and reduced model was conducted to test the effect of removing this set of items. 2-sided *P*-values of less than 0.05 were considered as statistically significant. The data were analyzed by using SPSS Version 20 (IBM SPSS Statistics, IBM Corporation, Armonk, NY).

## Results

A total of 1150 mother-newborn dyads were eligible and recruited during the study period. Among the 1150 participants, 972 were included in the final analysis after excluding 82 participants who could not be reached by phone and an additional 96 participants for whom there were incomplete data.

### Sample characteristics

Table 1 summarizes the characteristics of the 972 mother-newborn dyads.

**Table 1:** Maternal and newborn characteristics

Total	n = 972 (%)		
Maternal characteristics			
Education level		Pregnancy Complication	
Primary and under	83 (8)	None	584 (60)
Secondary	540 (56)	Maternal condition^b^	206 (21)
Matric^a^ or higher	349 (36)	Delivery condition^c^	182 (19)
Race		Number of children	
African	576 (59)	0	284 (29)
Mixed	379 (39)	1	291 (30)
White/Other	17 (2)	2	242 (25)
Medical Insurance		3+	155 (16)
No	958 (99)	Delivery type	
Yes	14 (1)	Vaginal	569 (59)
Travel time to local clinic		Elective C-section	247 (25)
>1 hour	507 (52)	Emergent C-section	156 (16)
<1 hour	455 (47)		
Unknown	10 (1)		
Maternal Income			
Salary/Wages/Grants	160 (16)		
No income	799 (82)		
Refuse to disclose	13 (1)		
Newborn characteristics			
Birth weight (grams)		Length of stay (hours)	
<2500	247 (25)	0–24	255 (26)
≥2500	725 (75)	25–48	209 (22)
Gestational Age (weeks)		49–72	218 (22)
34–36	197 (20)	73–96	168 (17)
37–39	594 (61)	>96	122 (13)
40–42	178 (18)	Planned Diet	
Unknown	3 (<1)	Breastmilk	911 (94)
		Formula	61 (6)
Outcomes			
Follow-up within 7 days of discharge			
Yes	794 (82)		
No	177 (18)		

^a^Matric: completion or graduation from secondary school

^b^Maternal condition: anemia, gestational diabetes, HIV, hypertension, preeclampsia

^c^Delivery condition: breech presentation, failure to progress, fetal distress, placental abruption, twin gestation

Of the mothers, 92% had at least some secondary school education, with 36% receiving their secondary school certificate or higher. A majority of mothers reported having no source of income (82%) and almost all endorsed not having medical insurance (99%). Only 21% of mothers had an existing pregnancy-related condition prior to delivery (gestational diabetes, hypertension, preeclampsia, anemia), while 19% had a delivery complication, the most common being fetal distress and breech presentation. Among newborns, 569 (59%) were born by vaginal delivery and 403 (41%) were born by caesarean section, with almost 40% of these surgeries considered to be emergent cases. About one-fourth (26%) of mothers with their newborns were discharged within 24 h after delivery, with almost half of the cohort (48%) being discharged within 48 h. The majority of mothers (69%) who delivered via normal vaginal delivery were discharged between 6 and 48 h, with the average length of stay being 46.3 h. Those mothers who delivered via caesarean section were typically discharged between 48 to 120 h (75%), with a mean (standard deviation) length of stay of 78.6 h (17.4).

### Follow-up characteristics and predictors

Eighty-two percent of newborns had an outpatient follow-up visit within seven days after discharge. Of these visits, 27% occurred within the first two days after hospital discharge and 62% occurred at 3–5 days after discharge. Demographic profiles of the mother-newborn dyads that followed up and those who did not were similar. Among those who had yet to have a follow-up visit, the primary reasons reported for not following up included maternal illness, the newborn was well and therefore did not need to be seen, and no money for transport to the local clinic (Table 2).

**Table 2:** Reasons given for not attending a newborn follow-up visit (multiple answers possible)

Reasons	*n* = 212
Maternal illness	68
Baby is healthy	61
No money for transport	54
Long queue at the clinic	10
No reason given	10
Not informed about follow-up	9

Newborns born less than 37 weeks were more likely to follow up after hospital discharge in bivariate and multivariable analysis (OR 1.67, 95% CI [1.07, 2.63]). Mothers who completed secondary school or achieved a higher level of education were more likely to bring their newborn for a follow-up visit in the first week after discharge compared to mothers who had some secondary school education or less (OR 1.47, 95% CI [1.02, 2.12]). There was no significant association between time of hospital discharge, including early discharges <48 h, and increased follow-up after hospital discharge. Number of previous children, type of delivery, pregnancy condition, newborn birth weight, travel time to the nearest clinic and maternal income were also not associated with differences in follow-up utilization (Table 3).

**Table 3:** Factors associated with newborn follow-up visit after hospital discharge within seven days

	Bivariate analysis			Multivariable analysis		
Variable	Odds Ratio	95% CI	*p*-value	Odds Ratio	95% CI	*p*-value
Maternal Characteristics						
Age (years)						
< 21	Reference					
21–30	0.76	[0.41, 1.39]	.369			
> 30	0.77	[0.41, 1.44]	.77			
Education						
Primary	0.90	[0.51, 1.59]	.722	0.89	[0.50, 1.57]	.682
Secondary	Reference					
Matric^a^ and higher	1.43	[0.99, 2.05]	.054	1.47	[1.02, 2.12]	.037
Maternal Income						
None	Reference					
Salary/Wages/Grants	0.96	[0.62, 1.48]	.841	–	–	–
Travel time to local clinic						
< 1 h	Reference					
≥ 1 h	0.82	[0.59, 1.15]	.252	–	–	–
Number of children						
0	Reference					
1	0.70	[0.45, 1.08]	.107	–	–	–
2	1.07	[0.66, 1.75]	.779	–	–	–
≥ 3	0.97	[0.53, 1.79]	.933	–	–	–
Pregnancy Complication						
None	Reference					
Maternal^b^	1.06	[0.71, 1.59]	.777	–	–	–
Delivery^c^	1.38	[0.87, 2.18]	.170	–	–	–
Delivery Type						
Vaginal	Reference					
Scheduled CS^d^	1.21	[0.81, 1.80]	.343	–	–	–
Emergency CS	0.98	[0.63, 1.54]	.944	–	–	–
Newborn characteristics						
Gestational age						
34–36 weeks	1.61	[1.03, 2.53]	.037	1.67	[1.07, 2.63]	.026
37–42 weeks	Reference					
Birth weight (grams)						
1800–2499	1.15	[0.79, 1.69]	.466	–	–	–
2500–4000	Reference					
> 4000	1.45	[0.64, 3.30]	.374	–	–	–
Sex (male)	1.02	[0.73, 1.41]	.903			
Length of stay (hours)						
0–24	1.12	[0.71, 1.77]	.620	–	–	–
25–48	1.03	[0.65, 1.66]	.889	–	–	–
49–72	Reference					
73–96	1.42	[0.84, 2.42]	.195	–	–	–
> 96	1.61	[0.87, 2.95]	.127	–	–	–

^a^Matric: completion or graduation from secondary school

^b^Maternal condition: anemia, gestational diabetes, HIV, hypertension, preeclampsia

^c^Delivery condition: breech presentation, failure to progress, fetal distress, placental abruption, twin gestation

^d^CS caesarean section

## Discussion

Our study shows that 4 in 5 newborns have a follow-up visit within seven days after discharge from a large tertiary hospital. The follow-up rate seen in this South African cohort is on the high end compared to other studies looking at newborn follow-up. In countries like the United States, follow-up rates varied depending on area and postnatal services available. Madlon-Kay and Ashe found an 84% newborn follow-up rate within two weeks after birth from a community hospital, but this included home visits as well as clinic visits, and no patient was discharged prior to 24 h [19]. Other studies have shown follow-up rates at 37% and 54% in the first 6–7 days after discharge [20, 21].

In many low- and middle-income countries (LMIC), the follow-up rate is generally much lower with rates of 8% over the first week of life to 23–65% over the first six weeks following birth in areas of Asia and Africa [22–25]. The prevalence of home births in many of these areas is high; thus, knowledge of and access to postnatal care may be different than in our study cohort, where all deliveries took place in a tertiary healthcare facility in an urban setting. The relatively high-risk maternal population and near-universal receipt of some level of antenatal care may have sensitized this group to the importance of seeking healthcare through the postnatal period. Indeed, receipt of antenatal care has been associated with increased postnatal care utilization [23, 24]. At the same time, multiple studies have found that mothers who deliver at a health facility may be less likely to attend postnatal care than those who delivered outside of a facility, as the assumption is the mother and newborn have already been evaluated by a healthcare provider [23, 25].

Variability in obtaining postnatal care exists despite recommendations by the World Health Organization, the American Academy of Pediatrics, and South Africa’s Maternity Guidelines Committee that newborn care should be obtained within the first few days of life, particularly if there is early discharge from a healthcare setting or a home delivery [8, 9, 15]. However, no significant difference was seen in follow-up rates among newborns discharged before 48 h and newborns discharged later. Reassuringly, late preterm newborns in our study were more likely to attend a follow-up visit after discharge than those born at term. The increased likelihood for follow-up among late preterm newborns is encouraging as they are a higher-risk group for readmission to the hospital [5].

Many studies have shown factors that affect utilization and attendance at postnatal visits in areas with low follow-up include lower income, lower maternal education level, multiparity, and living in a rural area [19, 23, 25, 26]. Among our study cohort, higher level of education did play a role in increased attendance with the first newborn visit after hospital discharge; however, no association was seen with parity, maternal income distribution was skewed preventing analysis, and living area was not assessed. Greater travel distances between the family and a health facility is associated with increased child mortality and decreased maternal utilization of services in LMIC [27]. More than half of our study population reported living more than 1 h from the nearest clinic, but travel time was not associated with decreased follow-up.

The major reasons for not seeking newborn follow-up care after discharge were that mothers thought it was unnecessary or had an issue with access, particularly the cost of getting to a local facility for care. These responses match barriers identified in many other LMIC where utilization of postnatal care services is low, namely, from lack of emphasis on the importance of the postnatal visit and concerns with access, including transportation costs and distance to the nearest healthcare facility [22, 28–31]. Interestingly enough, maternal health issues were the most common reason for failing to follow up at a health clinic, underscoring the importance of the first follow-up visit, which in most LMIC is typically a joint visit when both the mother’s and the newborn’s health are assessed and treated accordingly. Otherwise, most mothers in our cohort seemed to be aware of the existence of postnatal care services, as only a fraction reported not knowing about having to follow-up, which other studies have shown can be a significant hindrance to appropriate utilization of care [30, 31].

Although there was a surprisingly high rate of follow-up in our study population, there is still room for improvement. Currently, there are a handful of studies evaluating the efficacy of various strategies to improve follow-up rates. One strategy is to provide care coordination in the form of discharge follow-up appointments. Multiple studies have found a benefit to having an appointment date and time set prior to discharge to increase compliance with the first newborn visit [32, 33]. Primary care intake coordination to help with appointment and rescheduling no-shows after hospital discharge, as well as home nursing visits and phone call check-ins, have been shown to lead to increased compliance with and earlier timing of first follow-up visits [34–36]. Another option that has been employed in increasing health maintenance compliance is text message reminders [37]; a similar strategy for newborn follow-up could be beneficial. If health policy for newborn follow-up is to remain the same, stakeholders in the Western Cape Region might consider additional region-specific, culturally-competent strategies to increase follow-up rates.

The main strength of this study is the large cohort that was recruited and contacted for follow-up questioning. Limitations to the study included self-report of newborn follow-up, which could have introduced an element of reporting and/or self-selection bias. The mothers approached during recruitment may have been inclined to change their follow-up behavior because, through enrollment, they were sensitized to the need for proper newborn care and the advantages of timely follow-up. Our follow-up rate of over 80%, which is on the high side compared to other studies, lends support to the possibility of inadvertently influencing the study population. The study population was obtained through a convenience sample in a specific healthcare setting and may also be prone to selection bias; thus, it may not be possible to generalize our results to the rest of the South African population. The cohort had a different demographic distribution than the general population given Tygerberg Hospital’s catchment population and tertiary care status. Additionally, we chose to collect maternal income figures and analyze their association with newborn follow-up; it is possible that determining the household income of our study participants might have produced different results. Finally, our exclusion criteria (non-English-speaking, no cell phone contact) may have selected for a higher-educated, higher-income study population that would be more likely to follow up.

## Conclusions

Follow-up among newborns after hospital discharge from a referral center in the Western Cape region of South Africa was found to be comparatively high at 82%. No difference in follow-up rate was seen among those discharged at less than 48 h compared to those with longer lengths of stay. This finding suggests a continued need to further educate this potentially high-risk group on the importance of timely follow-up. Further research is needed to determine if lack of timely follow-up is associated with increased morbidity and mortality and to identify novel strategies to overcome barriers preventing appropriate newborn follow-up after hospital discharge.

## Abbreviations

LMIC: low- and middle-income countries
